# Parental Risk Factors among Children with Reactive Attachment Disorder Referred to Specialized Services: A Nationwide Population-Based Study

**DOI:** 10.1007/s10578-018-00861-6

**Published:** 2018-12-29

**Authors:** Subina Upadhyaya, Roshan Chudal, Terhi Luntamo, Jari Sinkkonen, Susanna Hinkka-Yli-Salomäki, Hitoshi Kaneko, Andre Sourander

**Affiliations:** 1 Department of Child Psychiatry, Research Center for Child Psychiatry, University of Turku, Lemminkäisenkatu 3/ Teutori 3rd Floor, 20540 Turku, Finland; 2 Psychological Support and Research Center for Human Development, Nagoya University, Nagoya, Japan; 3 Department of Child Psychiatry, Turku University Hospital, Turku, Finland

**Keywords:** Offspring reactive attachment disorder, Parental risk factors, Population-based study, Parental psychiatric disorders

## Abstract

This nationwide population-based register study examined the family and parental risk factors associated with offspring reactive attachment disorder (RAD). We identified 614 children diagnosed with RAD from the Finnish Care Register for Health Care and each case was matched with four controls. Univariate and multivariate models examined the associations between risk factors and RAD. In the multivariate model, offspring RAD was associated with only mother, only father and both parents having psychiatric diagnoses. Increased odds were observed for maternal smoking during pregnancy, single motherhood and paternal age ≥ 45 years. This study provides information on several parental adversities and offspring RAD that have important implications for public health, when planning early prevention and interventions in infant mental health.

## Introduction

Infants become attached to their primary caregiver and seek comfort and protection when they are distressed or threatened. This phenomenon is considered to be biologically pre-programmed and has emerged as a result of evolutionary pressures [1]. Bowlby defined attachment as a strong tendency to seek proximity with a primary caregiver, when frightened, tired or ill. The innate behaviors or social releasers from infants, such as crying and smiling, stimulate the caregiving behavior in adults. This need of physical proximity not only promotes the emotional security, but also ensures the infant’s protection and survival. Attachment develops during the first year of life, proceeding through various stages. Individual differences in attachment behavior can be assessed by strange situation procedure, developed by Ainsworth [2]. The continuous disruption of attachment between primary caregiver and infant may result in social and emotional difficulties [3]. The quality of attachment influences the child’s risk for psychiatric problems and disorders, either as a protective factor or as a risk factor [4, 5].

The International Statistical Classification of Diseases and Related Health Problems—10th Revision (ICD-10), which was published in 1992, separates attachment disorders into reactive attachment disorder (RAD) and disinhibited attachment disorder, while the Diagnostic and Statistical Manual of Mental Disorders—5th edition (DSM-5), published in 2013, separates attachment disorders into RAD and disinhibited social engagement disorder. The criteria for separating two distinct disorders were based on research in the last few decades that explained their differences in phenotypic characteristics, correlates, courses and responses to interventions [6, 7]. RAD is characterized by ambivalence about seeking comfort from a caregiver, emotional withdrawal, lack of social approach, reduced positive affect and unexplained fear or irritability. While children with RAD display limited or no positive effects, children with disinhibited attachment are intrusive, lack appropriate social and emotional boundaries and display socially disinhibited behavior with strangers [8, 9]. RAD needs to be distinguished from other psychopathologies that involve hypervigilance and behaviors associated with autism spectrum disorders. The differential diagnosis is often clarified by specific psychopathology to the pathological attachment and the fact that notable improvements occur when the individual is placed in a more sensitive, protective caregiving environment [10, 11].

Reactive attachment disorder has mostly been studied in high-risk populations. Higher prevalence have been observed in high-risk populations i.e., 1.40% of children in a deprived area of the United Kingdom [12], 19.4% of children in foster care [13] and 38–40% of maltreated toddlers in foster care [14]. Children who have experienced severe deprivation, neglect or insufficient caregiving face an increased risk of RAD [14, 15]. A study of twins in a general population suggested that harsh parenting behavior was associated with behaviors similar to attachment disorders among children and that these associations might be mediated by both environment and genetics [16]. However, only the Relationship Problems Questionnaire was used to measure the behavior in that cohort, instead of validated interviews or detailed diagnostic examinations. RAD has been associated with an increased risk of comorbidity, including emotional, behavioral and learning difficulties [13, 17]. A study of maltreated toddlers in foster care examined maternal risk factors for offspring RAD, including lack of education, teenage pregnancies, intimate partner violence, criminal history, depressed moods, maltreatment as a child, psychiatric history and a history of substance abuse. Of those risk factors, any maternal psychiatric history was associated with a 2.3-fold increased risk of offspring RAD [14].

To date, there has only been one nationwide population-based study on risk factors for attachment disorders [18]. That Danish register-based study examined mothers and fathers with a history of homelessness and offspring attachment disorders, adjusting for parental age at birth and parental psychiatric disorders. However, the study found that attachment disorders were not separated into two distinct disorders and no other parental risk factors were examined.

This is the first nationwide register-based study to examine specific parental psychiatric diagnoses associated with offspring RAD. In addition, it examines several potential risk factors, including maternal smoking during pregnancy, socioeconomic status (SES), marital status, living in an urban area and parental age. Based on previous clinical studies on risk factors for RAD, we hypothesized that parental psychopathology and other risk factors would be associated with an increased risk of offspring RAD.

## Methods

The Finnish perinatal study on RAD was a nationwide population-based study with a nested case control study design. The sampling population comprised 964,929 live singleton infants born in Finland between 1 January 1996 and 31 December 2012. Information on the cases and controls, and their parents, was collected from The Care Register for Health Care (Care Register), The Finnish Central Population Register (Population Register) and The Finnish Medical Birth Register (Birth Register).

The Care Register contains the patient’s personal identity code, date of birth, sex, the dates of any medical admissions and discharges and any primary diagnoses at discharge, along with three subsidiary diagnoses. The Population Register contains basic information about Finnish citizens and permanent residents in Finland. It includes their name, personal identity code, address, citizenship, native language, date of birth and date of death, if applicable. The Birth Register provides information on demographic characteristics. It contains comprehensive and standardized information for all births in Finland, covering the pregnancy, the prenatal period and the neonatal period up to age of 7 days. Information regarding the place of birth, the mother’s marital status, maternal smoking and maternal SES are also included in this register. These registers were linked together using the unique personal identity codes that have been provided to all Finnish residents since 1964. These registers, and how they link together, have previously been described [19–22].

### Diagnosing RAD in Finland

Health care services in Finland are publicly funded and all children visit child health clinics for at least 15 periodic check-ups between birth and the age of seven. Most of the visits take place during the child’s first year of life. These check-ups are led by registered nurses and general practitioners, who can consult psychologists and social workers, if necessary. The clinics monitor, support and document the child’s physical, psychological and social development. More than 99.5% of families with children use these services [23]. After each visit, health care personnel document the child’s life circumstances, as well as their physical and psychosocial development. They also record the information about child’s family, day care or school. If a child is suspected of RAD or more unspecific problems of social functioning, a referral is made to specialized mental health services. There are 20 district hospitals and five university hospitals who provide specialized mental health services in Finland [24] and one in eight children from birth to 14 years visit specialized services for psychiatric or neurodevelopmental disorders [25]. The Finnish health care system draws on all information it can, when assessing children and these include the child, the family, daycare, schools and social services. They also examine the history of health check-ups, parent–child relationships and life events. Diagnostic information in the national registers are based on clinical diagnoses and they were recorded into the registers according to ICD-10 criteria. All diagnoses are routinely registered in the Care Register by healthcare personnel.

In Finland, any diagnosis of RAD is performed in a child psychiatric clinic by a team of professionals including a child psychiatrist, psychologist and a social worker. The child is assessed together with primary caregivers, as well as individually, by several professionals and the findings are discussed by the team. Comprehensive interviews with parents, and often with personnel from the child’s daycare facility or school are conducted. In some cases, standardized tools, such as the Marschak Interaction Method [26, 27], the Parent Development Interview [28] and the Bayley Scales of Infant and Toddler Development [29] are used to assess parent–child interaction, including separation and reunion, and the child’s cognitive development. Thus, the child is observed in different surroundings, together with peers and adults.

### Study Participants

#### Case Identification

We identified all live singleton infants born in Finland from 1 January 1996 to 31 December 2012 and recorded in the Care Register with a diagnosis of RAD, with the ICD-10 code F94.1. Children diagnosed with severe or profound mental retardation (F72 and/or F73) and pervasive developmental disorders (F84) were excluded. A total of 614 cases were identified.

#### Control Identification

The controls in this study were live singleton infants born in Finland during the same time period, but without any diagnosis of RAD in the Care Register or severe or profound mental retardation (F72 and/or F73), pervasive developmental disorders (F84) or anxiety disorder (F 40–42 and/or F93). Each case was matched with four controls based on their date of birth (± 30 days) and sex. A total of 2423 controls were identified.

Children with severe or profound mental retardation were excluded among cases and controls because their illness would have made it difficult to assess RAD symptoms. Children with pervasive developmental disorders (PDD) were excluded to increase the reliability of RAD diagnoses, as PDD is an exclusion criterion for diagnosing RAD, according to ICD-10 [9]. Children with PDD display similar characteristics to RAD and, as a result, they could be misdiagnosed with RAD in the early years of life. Children with anxiety disorders were not included in the control group as this study was a sub-study of a larger sample of anxiety and trauma related disorders. Therefore children with anxiety disorders were not included in the initial sample.

### Demographic Factors

Information on demographics were obtained from the Birth Register. Maternal age was divided into six categories (≤ 19, 20–24, 25–29, 30–34, 35–39, ≥ 40) and paternal age was divided into seven categories (≤ 19, 20–24, 25–29, 30–34, 35–39, 40–44 and ≥ 45). Maternal SES was categorized into upper white collar workers, lower white collar workers, blue collar workers and other workers. The classification of maternal SES was primarily based on occupational status. Upper white collar workers included upper clerical workers, such as managers, experts in their field and teachers. Lower white collar workers included lower clerical workers such as people doing office work who did not fall into the previous category. Blue collar workers included those with manual jobs and others included people running their own businesses and people who were not engaged in the labor force, such as students, homemakers and unemployed people. Women who reported they were in education, rather than doing a job, were classified as upper white collar workers if they graduated from university and lower white collar workers if they had a vocational degree, which was lower than a university degree. Marital status was categorized as either single or married/in a relationship and residence was categorized as urban, semi-urban or rural.

### Lifestyle Related Factors

Information on maternal smoking was obtained from the Birth Register and dichotomized as yes or no.

### Parental Psychiatric Diagnoses

Information on parental psychiatric diagnoses were derived from Care Register. Parents were followed up from January 1969 to December 2012. We used two different kinds of categorization for parental psychiatric diagnosis. First, parental psychiatric diagnoses were aggregated and categorized as only mother, only father, both the mother and father and no psychiatric diagnoses. This categorization was included with other risk factors in the multivariate analyses. Second, maternal and paternal adulthood-onset psychiatric disorders were examined separately in the following categories as present (score of 1) or absent (score of 0): schizophrenia and schizoaffective disorders; other psychoses; bipolar disorders; depression; anxiety disorders; personality disorders and alcohol and drug addiction/abuse. If a parent was diagnosed with schizophrenia or a schizoaffective disorder, he or she would not be assigned to any of the other categories. This hierarchical model was applied because schizophrenia and schizoaffective disorders are distinctly severe chronic conditions that cause impairment. In other cases, a parent could be assigned to several diagnostic categories. A similar method of categorization has been used in previous studies [30, 31]. In addition, different combinations of maternal and paternal adulthood-onset psychiatric disorders were also studied.

### Psychiatric Comorbidity

The Care Register was used to obtain all psychiatric diagnoses among RAD cases according to the following ICD-10 codes. The comorbidities with RAD were examined for: attention deficit hyperactivity disorder (F90), emotional disorders with onset specific to childhood (F93), learning and coordination disorders (F80–F83), conduct and oppositional disorders (F91–F92), tic disorders (F95), elective mutism (F94.0), disinhibited attachment disorder of childhood (F94.2), depression (F32, F33, F34, F38, F39), obsessive compulsive disorder (F42) and reaction to severe stress and adjustment disorders (F43).

### Statistical Analysis

Univariate analysis using conditional logistic regression was used to examine the associations between potential risk factors, including parental psychopathology, maternal smoking, maternal age, paternal age, maternal SES, marital status and residence and offspring RAD. The variables shown to be significantly associated with offspring RAD in the univariate analyses were selected for the multivariate analyses using conditional logistic regression. The limit of significance for selecting variables for the multivariate analyses was *p* < 0.1. The association between offspring RAD to the exposure was reported as an odds ratio (OR) and two-sided 95% confidence interval (95% CI). The level of significance was *p* < 0.05.

Maternal and paternal adulthood-onset psychiatric diagnoses were examined separately as present or absent for schizophrenia and schizoaffective disorders, other psychoses, bipolar disorders, depression, anxiety disorders, personality disorders and alcohol and drug addiction/abuse. The association between each maternal and paternal adulthood-onset psychiatric disorder, and RAD was adjusted for the potential risk factors that were shown to be significantly associated with offspring RAD in the multivariate analysis (*p* < 0.1). Conditional logistic regression analyses were used and the associations were reported as odds ratios (OR) and two-sided 95% confidence intervals (95% CI). The level of significance was *p* < 0.05.

In the additional analyses, the frequencies of different combinations of maternal and paternal adulthood-onset psychiatric diagnoses were examined among the cases and controls and were reported as percentages.

The RAD cases were stratified into two groups: RAD with any comorbid psychiatric diagnoses (at least one comorbid psychiatric disorder) and RAD without any comorbid psychiatric diagnoses. The conditional logistic regression analyses were used to examine the associations between parental risk factors and each stratified group. The results were reported as ORs and two-sided 95% CIs. The level of significance was *p* < 0.05.

The statistical analyses were performed with SAS statistical software, version 9.4 (SAS Institute Inc, Cary, NC, USA).

## Results

The mean age at diagnosis of RAD was 7.36 years, with a standard deviation (*SD*) of 3.0 years and a range of 0–16 years. Of the 614 cases, 61% were male. The cumulative incidence of RAD cases treated in specialized health care services over the 16-year period (1996–2012) was 6.38/10,000 births: 7.38/10,000 among males and 4.92/10,000 among females.

Table 1 shows the univariate and multivariate models for the risk factors. Increased odds for RAD were associated with a maternal diagnosis of a psychiatric disorder (OR 8.82, 95% CI 5.87–13.26) and a paternal diagnosis of a psychiatric disorder (OR 5.60, 95% CI 3.56–8.82). Similarly, the odds for the child having RAD were significantly increased when both parents were diagnosed with any psychiatric disorders (OR 51.47, 95% CI 31.50-84.11) when compared to only mothers (*p* < 0.001) or only fathers (*p* < 0.001). Increased odds of having RAD were also associated with maternal smoking during pregnancy (OR 2.34, 95% CI 1.67–3.28), single motherhood (OR 2.18, 95% CI 1.29–3.66) and advanced paternal age (OR 2.85, 95% CI 1.32–6.14).

**Table 1:** Univariate and multivariate models for the association between parental risk factors and RAD

	Cases (*n* = 614) *n* (%)	Controls (*n* = 2423) *n* (%)	Univariate OR (95% CI)	*P* value	Multivariate OR (95% CI)	*P* value
Psychopathology						
Only mothers	181 (29.48)	254 (10.48)	16.80 (11.93–23.65)	< 0.001	8.82 (5.87–13.26)	< 0.001
Only fathers	83 (13.52)	199 (8.21)	9.19 (6.26–13.48)	< 0.001	5.60 (3.56–8.82)	< 0.001
Both	268 (43.65)	70 (2.89)	86.05 (56.75-130.48)	< 0.001	51.47 (31.50-84.11)	< 0.001
None	82 (13.36)	1,900 (78.42)	Reference		Reference	
Maternal smoking^a^						
Yes	328 (55.13)	351 (14.97)	6.72 (5.46–8.27)	< 0.001	2.34 (1.67–3.28)	< 0.001
No	267 (44.87)	1,994 (85.03)	Reference		Reference	
Maternal SES						
Upper white collar workers	26 (4.23)	398 (16.43)	Reference		Reference	
Lower white collar workers	128 (20.85)	1,014 (41.85)	1.93 (1.24-3.00)	0.003	0.91 (0.49–1.67)	0.760
Blue collar workers	134 (21.82)	371 (15.31)	5.86 (3.73–9.20)	< 0.001	1.84 (0.96–3.52)	0.063
Others	200 (32.57)	439 (18.12)	7.36 (4.74–11.43)	< 0.001	1.36 (0.70–2.66)	0.360
Unknown	126 (20.52)	201 (8.30)	10.48 (6.57–16.73)	< 0.001	2.62 (1.30–5.33)	0.007
Marital status						
Single	115 (18.73)	104 (4.29)	6.07 (4.51–8.18)	< 0.001	2.18 (1.29–3.66)	0.003
Married/in a relationship	392 (63.84)	2,138 (88.24)	Reference		Reference	
Unknown	107 (17.43)	181 (7.47)	3.19 (2.44–4.17)	< 0.001	1.41 (0.88–2.26)	0.148
Residence^b^						
Urban	438 (71.68)	1,542 (63.90)	1.54 (1.20–1.98)	< 0.001	1.49 (0.99–2.25)	0.053
Semi-urban	85 (13.91)	392 (16.24)	1.18 (0.84–1.64)	0.326	1.23 (0.72–2.11)	0.443
Rural	88 (14.40)	479 (19.85)	Reference		Reference	
Maternal age						
≤ 19	81 (13.18)	68 (2.81)	5.83 (4.02–8.45)	< 0.001	1.59 (0.76–3.30)	0.214
20–24	178 (28.99)	396 (16.34)	2.36 (1.83–3.04)	< 0.001	1.49 (0.92–2.40)	0.100
25–29	141 (22.96)	731 (30.17)	Reference		Reference	
30–34	113 (18.40)	769 (31.74)	0.76 (0.58–0.99)	0.043	0.91 (0.57–1.43)	0.684
35–39	78 (12.70)	363 (14.98)	1.15 (0.84–1.56)	0.373	0.88 (0.49–1.56)	0.662
≥ 40	23 (3.75)	96 (3.96)	1.18 (0.72–1.93)	0.498	0.61 (0.25–1.47)	0.270
Paternal age^c^						
≤ 19	25 (4.48)	27 (1.12)	3.85 (2.12–6.98)	< 0.001	0.85 (0.28–2.57)	0.772
20–24	118 (21.15)	222 (9.23)	2.30 (1.71–3.08)	< 0.001	0.84 (0.50–1.40)	0.509
25–29	143 (25.63)	647 (26.89)	Reference		Reference	
30–34	99 (17.74)	775 (32.21)	0.58 (0.44–0.77)	< 0.001	0.67 (0.42–1.07)	0.095
35–39	87 (15.59)	479 (19.91)	0.80 (0.60–1.08)	0.143	0.97 (0.57–1.65)	0.925
40–44	42 (7.53)	174 (7.23)	1.10 (0.74–1.62)	0.641	1.16 (0.56–2.40)	0.688
≥ 45	44 (7.89)	82 (3.41)	2.45 (1.61–3.72)	< 0.001	2.85 (1.32–6.14)	0.007

*OR* odds ratio, *CI* confidence interval, *SES* socioeconomic status

^a^19 Cases and 78 controls missing

^b^3 Cases and 10 controls missing

^c^56 Cases and 17 controls missing

As shown in Table 2, all the psychiatric disorders in both the mothers and fathers were associated with offspring RAD, namely schizophrenia and schizoaffective disorders, psychoses, bipolar disorder, depression, anxiety disorder, personality disorder and alcohol and drug addiction/abuse. The strength of the associations between parental psychopathology and offspring RAD is highlighted by the fact that 40.39% of the mothers of cases were diagnosed with depression (controls 7.22%), 19.38% with anxiety disorders (controls 2.97%), 22.48% with personality disorders (controls 1.20%) and 34.20% with alcohol and drug addiction/abuse (controls 1.65%). Similarly, among the fathers of cases, 24.19% had depression (controls 4.32%), 10.04% had anxiety disorders (controls 2.29%), 13.62% had personality disorders (controls 1.58%) and 38.71% had alcohol and drug addiction/abuse (controls 3.45%).

**Table 2:** Unadjusted and adjusted association for parental specific psychopathology and RAD

	Cases (*n* = 614) *n* (%)	Controls (*n* = 2423) *n* (%)	Unadjusted OR (95% CI)	*P* value	Adjusted^a^ OR (95% CI)	*P* value
Schizophrenia and schizoaffective						
Maternal	44 (7.17)	7 (0.29)	24.63 (11.09–54.70)	< 0.001	13.17 (5.37–32.30)	< 0.001
Paternal	20 (3.58)	7 (0.29)	11.20 (4.73–26.50)	< 0.001	8.64 (3.08–24.25)	< 0.001
Other psychoses						
Maternal	46 (7.49)	13 (0.54)	14.86 (7.87–28.08)	< 0.001	12.18 (5.18–28.60)	< 0.001
Paternal	28 (5.02)	13 (0.54)	9.14 (4.65–18.00)	< 0.001	7.45 (3.13–17.73)	< 0.001
Bipolar disorder						
Maternal	42 (6.84)	16 (0.66)	11.62 (6.34–21.31)	< 0.001	8.96 (4.12–19.48)	< 0.001
Paternal	18 (3.23)	14 (0.58)	5.78 (2.77–12.02)	< 0.001	5.05 (1.89–13.51)	0.001
Depression						
Maternal	248 (40.39)	175 (7.22)	9.21 (7.19–11.80)	< 0.001	7.46 (5.38–10.35)	< 0.001
Paternal	135 (24.19)	104 (4.32)	6.60 (4.95–8.80)	< 0.001	5.05 (3.54–7.21)	< 0.001
Anxiety disorders						
Maternal	119 (19.38)	72 (2.97)	7.90 (5.73–10.90)	< 0.001	5.65 (3.71–8.61)	< 0.001
Paternal	56 (10.04)	55 (2.29)	4.60 (3.10–6.83)	< 0.001	3.48 (2.13–5.70)	< 0.001
Personality disorders						
Maternal	138 (22.48)	29 (1.20)	25.07 (15.83–39.71)	< 0.001	17.93 (9.98–32.18)	< 0.001
Paternal	76 (13.62)	38 (1.58)	8.77 (5.82–13.22)	< 0.001	6.0 (3.62–9.90)	< 0.001
Alcohol and drug addiction/abuse						
Maternal	210 (34.20)	40 (1.65)	30.69 (20.41–46.16)	< 0.001	18.07 (10.90–29.97)	< 0.001
Paternal	216 (38.71)	83 (3.45)	17.28 (12.51–23.86)	< 0.001	8.81 (6.07–12.78)	< 0.001

*OR* odds ratio, *CI* confidence interval

^a^Adjusted for marital status, urbanity, maternal smoking, SES and paternal age [*p* < 0.1 in multivariate model (Table 1)]

In the additional analysis (Table 3), different combinations of maternal and paternal psychopathology were examined. As shown in this table, 20.03% of those with RAD had both parents diagnosed with alcohol and drug addiction/abuse (controls 0.25%), 17.43% had mothers diagnosed with depression and fathers with alcohol and drug addiction/abuse (controls 0.66%) and 10.75% had both parents diagnosed with depression (controls 0.83%).

**Table 3:** Frequencies of different combinations of maternal and paternal psychopathology

	Maternal							
	Schizophrenia		Psychoses		Bipolar disorder		Depression	
	Cases (*n*, %)	Controls (*n*, %)	Cases (*n*, %)	Controls (*n*, %)	Cases (*n*, %)	Controls (*n*, %)	Cases (*n*, %)	Controls (*n*, %)
Paternal								
Schizophrenia	4 (0.65)	0 (0)	8 (1.30)	1 (0.04)	0 (0)	0 (0)	7 (1.14)	1 (0.04)
Psychoses	7 (1.14)	0 (0)	11 (1.79)	1 (0.04)	4 (0.65)	0 (0)	19 (3.09)	5 (0.21)
Bipolar disorder	0 (0)	0 (0)	2 (0.33)	1 (0.04)	3 (0.49)	0 (0)	9 (1.47)	4 (0.17)
Depression	7 (1.14)	0 (0)	17 (2.77)	1 (0.04)	13 (2.12)	1 (0.04)	66 (10.75)	20 (0.83)
Anxiety disorders	2 (0.33)	0 (0)	5 (0.81)	1 (0.04)	3 (0.49)	1 (0.04)	24 (3.91)	8 (0.33)
Personality disorders	3 (0.49)	0 (0)	13 (2.12)	1 (0.04)	6 (0.98)	0 (0)	40 (6.51)	5 (0.21)
Alcohol and drug addiction/abuse	15 (2.44)	0 (0)	31 (5.05)	2 (0.08)	17 (2.77)	0 (0)	107 (17.43)	16 (0.66)
No any psychiatric diagnosis	65 (10.58)	23 (0.95)	81 (13.19)	32 (1.32)	75 (12.21)	32 (1.32)	139 (22.63)	147 (6.06)

	Maternal							
	Anxiety disorders		Personality disorders		Alcohol and drug addiction/abuse		No any psychiatric diagnosis	
	Cases (*n*, %)	Controls (*n*, %)	Cases (*n*, %)	Controls (*n*, %)	Cases (*n*, %)	Controls (*n*, %)	Cases (*n*, %)	Controls (*n*, %)
Paternal								
Schizophrenia	2 (0.33)	0 (0)	7 (1.14)	0 (0)	5 (0.81)	0 (0)	4 (0.65)	5 (0.20)
Psychoses	9 (1.47)	1 (0.04)	11 (1.79)	0 (0)	12 (1.95)	1 (0.04)	9 (1.46)	12 (0.49)
Bipolar disorder	6 (0.98)	0 (0)	3 (0.49)	1 (0.04)	7 (1.14)	0 (0)	10 (1.62)	11 (0.45)
Depression	35 (5.70)	8 (0.33)	44 (7.17)	4 (0.17)	68 (11.07)	5 (0.21)	33 (5.37)	78 (3.22)
Anxiety disorders	12 (1.95)	2 (0.08)	11 (1.79)	1 (0.04)	28 (4.56)	1 (0.04)	16 (2.60)	44 (1.81)
Personality disorders	19 (3.09)	3 (0.12)	25 (4.07)	2 (0.08)	38 (6.19)	4 (0.17)	20 (3.25)	30 (1.23)
Alcohol and drug addiction/abuse	60 (9.77)	7 (0.29)	62 (10.10)	2 (0.08)	123 (20.03)	6 (0.25)	51 (8.30)	58 (2.39)
No any psychiatric diagnosis	91 (14.82)	73 (3.01)	100 (16.28)	39 (1.61)	104 (16.93)	43 (1.77)	138 (22.47)	1917 (79.11)

No diagnostic hierarchy was followed in parental psychiatric diagnoses and a single patient could have been included in several diagnostic categories. Cases (*N* = 614); Controls (*N* = 2423)

A total of 484 (78.82%) cases had at least one comorbid psychiatric diagnoses. The most common comorbid diagnoses were conduct and oppositional disorders (35.34%), learning and coordination disorders (29.64%), attention deficit hyperactivity disorder (23.94%), emotional disorders with onset specific to childhood (21.66%), reaction to severe stress and adjustment disorders (15.80%) and depression (10.75%). When we stratified the RAD cases into RAD with any comorbid psychiatric diagnoses (at least one comorbid psychiatric disorder) and RAD without any comorbid psychiatric diagnoses, the overall associations between parental risk factors and each stratified group remained significant, except residence (data available on request).

## Discussion

This study had three main findings. First, it showed strong associations between parental psychopathology and offspring RAD, in particular increased odds of RAD when both parents had a psychiatric diagnosis. Second, smoking during pregnancy was associated with offspring RAD. Third, single motherhood and advanced parental age were independently associated with RAD.

Both maternal and paternal psychiatric diagnoses were associated with increased risks of offspring RAD. The risk of being diagnosed with RAD was particularly high when both parents had a psychiatric diagnosis. These findings support similar clinical observations in an earlier non population-based sample [14]. Findings from a twin study suggested the influence of both genes and environment in the development of attachment disorder behavior [16]. Secure attachment requires predictability and sensitivity to the child’s needs [32] and parental psychopathology has been associated with insecure attachment in children [33]. For example, parental depression has been associated with both withdrawn and intrusive parental behavior [34] and insensitive parenting, particularly neglectful or abusive caregiving. It has also has been associated with poor regulation of infant physiology and disturbances in development related to social behavior and threat processing [35, 36]. These negative effects may become stronger when both parents have psychiatric disorders than when a single parent has a psychiatric disorder, as the other parent can compensate for the affected partner’s difficulties.

In our study, the most common psychiatric diagnoses in parents with children with RAD were alcohol and drug addiction/abuse and depression. There are several possible mechanisms that could explain the association with parental substance use, including effects on the infants’ development, either through direct exposure in utero [37] or harsh parenting behavior [38]. For example, one study reported that the children of parents who abused substances were more likely to experience physical and sexual abuse [39] and, therefore, might be exposed to foster care and institutional rearing or repeated changes in caregiving behavior. Other studies found that RAD was more likely to be detected among children living in adverse caregiving environments or with severe deprivation [14, 15]. In addition, assortative mating has been shown to increase the likelihood that a mother with a particular psychiatric illness will have a spouse who also has a psychiatric disorder or a family history of psychiatric illness [40]. These findings add to the existing evidence about the non-specificity of early childhood adversity and subsequent child psychopathology [41].

The effect of maternal smoking persisted after adjusting for other risk factors, suggesting that smoking had an independent effect. Women who smoke heavily during pregnancy have been reported to have had a weaker attachment to their fetus [42]. It is likely that mothers who feel less attached to their growing baby are unable to regulate their smoking behavior because they lack motivation. In a Finnish study, children were significantly more likely to end up in foster care if their mothers smoked during pregnancy than if their mothers did not smoke [43]. There are several possible biological mechanisms that can underlie the effects of maternal smoking. Research on animals has suggested that prenatal nicotine exposure causes structural alterations in the brain reward system and leads to lower levels of dopamine being released into the ventral striatum [44, 45]. Similar findings have been observed in adolescents with prenatal exposure to maternal cigarette smoking [46]. Reduced activation in the ventral striatum has also been observed in children who were diagnosed with RAD and had a history of severe maltreatment [47]. Furthermore, maternal smoking during pregnancy has been associated with a reduction in the volume of cortical gray matter in children [48], which has also been observed in children with RAD and children with a history of severe maltreatment [49]. Thus, it is not clear whether these findings were due to early adversity or the RAD phenotype. On the other hand, our findings may also be due to the likelihood that parental psychopathology has been associated with higher rates of smoking. Although we adjusted maternal smoking for maternal psychopathology, it is possible that less severe parental psychiatric diagnoses could have been underdiagnosed and may have contributed to this association.

The specific association that we discovered between advanced paternal age and offspring RAD has not previously been reported. It has been suggested that mechanisms related to de novo germline mutation [50, 51] and epigenetic alterations [52] may underlie the effect that paternal age has on psychiatric disorders in their offspring. The inherited traits of parents with limited social interaction skills might result in delayed marriage and late fatherhood [53].

Our study had several limitations that need to be considered when interpreting the results. First, the mean age of the child that were diagnosed with RAD was 7.3 years. The RAD cases in this study were based on clinical diagnoses by specialized mental health services, meaning that there was usually a significant delay in getting a diagnosis. This was in line with our previous study, which was based on national registers and showed a delay of several years in getting a diagnosis of autism spectrum disorder [54]. Second, we used national registers to identify cases with RAD. Standardized interviews or scales designed and validated for diagnosing RAD among children would be more reliable than clinical diagnoses registered in the Care Register. However, although there are no uniformly recommended standardized interviews or scales to diagnose RAD in Finland, national registry diagnoses tend to be reliable in countries, like ours, which provide uniform training in psychiatry [55]. Third, because this was a register-based study, it did not completely represent all the cases of RAD in the Finnish population. It only covered the cases that used specialized services and these may have been the more severe cases. Likewise, RAD cases treated in private health clinics were not recorded in the Care Register and children who did not use any public health services would not have been included in our study. Therefore, we may have missed a number of undiagnosed or less severe cases. The control group may have included undiagnosed cases and that would have diminished the strength of the association. However, in Finland, all children visit child health clinics at least once a year before they start school at the age of seven. This increases the likelihood that a child with moderate or severe symptoms of psychiatric illness will be referred to specialized services for diagnostic assessment and registered in the Care Register. Furthermore, health care services in Finland are universal, they are financed by the state or municipalities and children are exempt from the generally low patient fees. Thus, it is likely that the public health care system would have had contact with most of the children with RAD. Fourth, we did not have access to information about insufficient child care and child custody. In Finland, parents with alcohol and/or substance abuse were significantly more likely to have their children placed in early and long-term custody [56]. RAD is more likely to be detected among children with severe deprivation or living in adverse caregiving environments [15]. Therefore, the availability of this information would have provided a much clearer perspective of the parental characteristics associated with an increased risk of RAD. Finally, children with anxiety disorders were not included in the control group as this study was a sub-study of a larger sample of anxiety and trauma related disorders. Therefore children with anxiety disorders were not included in the initial sample. However, children with RAD might also have emotional symptoms that are similar to anxiety, including hopelessness, apathy, being afraid, withdrawal, ambivalence, and mood changes.

## Summary

This study provides information on several novel findings on parental adversities and offspring RAD that have important implications when planning early prevention and interventions in infant mental health. Parental psychopathology had an exceptionally strong association with offspring RAD and this was much higher than the levels previously demonstrated by studies based on Finnish registers on other childhood psychiatric disorders, such as autism [57] and attention deficit hyperactivity disorder [30]. The risk for offspring RAD was higher when both parents had a psychiatric disorder. The mechanisms behind these associations can be explained by genetic factors, adversities related to parenting and parental risk behavior exposing the fetal brain to substance use. It is likely that these factors interact with, and strengthen, each other. As secure attachment during early childhood is considered to be one of the key elements for good mental health later in life, it is important to identify and help parents with psychiatric problems to develop a positive environment for their child as early as the antenatal period.

## References

[1] Bowlby J (1969). Attachment and loss: attachment.

[2] Ainsworth MDS, Blehar MC, Waters E, Wall S (1978). Patterns of attachment.

[3] Bowlby J (1973). Attachment and loss, vol2. Separation: anxiety and anger.

[4] Green J, Goldwyn R (2002). Annotation: attachment disorganisation and psychopathology: new findings in attachment research and their potential implications for developmental psychopathology in childhood. J Child Psychol Psychiatry.

[5] Zeanah CH, Berlin LJ, Boris NW (2011). Practitioner review: clinical applications of attachment theory and research for infants and young children. J Child Psychol Psychiatry.

[6] Zeanah CH, Gleason MM (2015). Annual Research Review: attachment disorders in early childhood – clinical presentation, causes, correlates, and treatment. J Child Psychol Psychiatry.

[7] Zeanah CH, Chesher T, Boris NW (2016). Practice parameter for the assessment and treatment of children and adolescents with reactive attachment disorder and disinhibited social engagement disorder. J Am Acad Child Adolesc Psychiatry.

[8] American Psychiatric Association (2013). Diagnostic and statistical manual of mental disorders.

[9] World Health Organization (1992). The ICD-10 classification of mental and behavioural disorders: clinical descriptions and diagnostic guidelines.

[10] Humphreys KL, Nelson CA, Fox NA, Zeanah CH (2017). Signs of reactive attachment disorder and disinhibited social engagement disorder at age 12 years: effects of institutional care history and high-quality foster care. Dev Psychopathol.

[11] Smyke AT, Zeanah CH, Fox NA, Nelson CA, Guthrie D (2010). Placement in foster care enhances quality of attachment among young institutionalized children. Child Dev.

[12] Minnis H, Macmillan S, Pritchett R, Young D, Wallace B, Butcher J, Sim F, Baynham K, Davidson C, Gillberg C (2013). Prevalence of reactive attachment disorder in a deprived population. Br J Psychiatry.

[13] Lehmann S, Havik OE, Havik T, Heiervang ER (2013). Mental disorders in foster children: a study of prevalence, comorbidity and risk factors. Child Adolesc Psychiatry Mental Health.

[14] Zeanah CH, Scheeringa M, Boris NW, Heller SS, Smyke AT, Trapani J (2004). Reactive attachment disorder in maltreated toddlers. Child Abuse Negl.

[15] Boris NW, Hinshaw-Fuselier SS, Smyke AT, Scheeringa MS, Heller SS, Zeanah CH (2004). Comparing criteria for attachment disorders: establishing reliability and validity in high-risk samples. J Am Acad Child Adolesc Psychiatry.

[16] Minnis H, Reekie J, Young D, O’Connor T, Ronald A, Gray A, Plomin R (2007). Genetic, environmental and gender influences on attachment disorder behaviours. Br J Psychiatry.

[17] Raaska H, Elovainio M, Sinkkonen J, Matomäki J, Mäkipää S, Lapinleimu H (2012). Internationally adopted children in Finland: parental evaluations of symptoms of reactive attachment disorder and learning difficulties—FINADO study. Child Care Health Dev.

[18] Nilsson SF, Laursen TM, Hjorthøj C, Thorup A, Nordentoft M (2017). Risk of psychiatric disorders in offspring of parents with a history of homelessness during childhood and adolescence in Denmark: a nationwide, register-based, cohort study. Lancet Public Health.

[19] Lampi KM, Banerjee PN, Gissler M, Hinkka-Yli-Salomäki S, Huttunen J, Kulmala U, Lindroos J, Niemelä S, Rihko M, Ristkari T, Saanakorpi K, Sarlin T, Sillanmäki L, McKeague IW, Surcel H, Helenius H, Brown AS, Sourander A (2011). Finnish prenatal study of autism and autism spectrum disorders (FIPS-A): overview and design. J Autism Dev Disord.

[20] Joelsson P, Chudal R, Gyllenberg D, Kesti A, Hinkka-Yli-Salomäki S, Virtanen J, Huttunen J, Ristkari T, Parkkola K, Gissler M, Sourander A (2016). Demographic characteristics and psychiatric comorbidity of children and adolescents diagnosed with ADHD in specialized healthcare. Child Psychiatry Hum Dev.

[21] Leivonen S, Voutilainen A, Hinkka-Yli-Salomäki S, Timonen-Soivio L, Chudal R, Gissler M, Huttunen J, Sourander A (2014). A nationwide register study of the characteristics, incidence and validity of diagnosed Tourette syndrome and other tic disorders. Acta Paediatr.

[22] Chudal R, Sucksdorff D, Suominen A, Lehti V, Hinkka-Yli-Salomäki S, Huttunen J, Ristkari T, Gissler M, McKeague IW, Brown AS, Sourander A (2014). Finnish prenatal study of bipolar disorders (FIPS-B): overview, design and description of the sample. Nord J Psychiatry.

[23] National Institute for Health and Welfare (2014) Perinatal statistics: parturients, deliveries and newborns 2014. http://www.julkari.fi/handle/10024/126971. Accessed 12 August 2018

[24] Vuorenkosky L, Mladovsky P, Mossialos E (2008). Finland: health system review. Health Syst Transit.

[25] Gyllenberg D, Gissler M, Malm H, Artama M, Hinkka-Yli-Salomäki S, Brown AS, Sourander A (2014). Specialized service use for psychiatric and neurodevelopmental disorders by age 14 in Finland. Psychiatr Serv.

[26] Hitchcock DL, Ammen S, O’Connor K, Backman TL (2008). Validating the Marschak interaction method rating system with adolescent mother-child dyads. Int J Play Ther.

[27] Martin EE, Snow MS, Sullivan K (2008). Patterns of relating between mothers and preschool-aged children using the Marschak interaction method rating system. Early Child Dev Care.

[28] Aber L, Slade A, Berger B, Bresgi I, Kaplan M (1985). The parent development interview.

[29] Bayley N (2006). Bayley scales of infant and toddler development: administration manual.

[30] Joelsson P, Chudal R, Uotila J, Suominen A, Sucksdorff D, Gyllenberg D, Sourander A (2017). Parental psychopathology and offspring attention-deficit/hyperactivity disorder in a nationwide sample. J Psychiatr Res.

[31] Leivonen S, Scharf JM, Mathews CA, Chudal R, Gyllenberg D, Sucksdorff D, Suominen A, Voutilainen A, Brown AS, Sourander A (2017). Parental psychopathology and tourette syndrome/chronic tic disorder in offspring: a nationwide case-control study. J Am Acad Child Adolesc Psychiatry.

[32] McGoron L, Gleason MM, Smyke AT, Drury SS, Nelson CA, Gregas MC, Fox NA, Zeanah CH (2012). Recovering from early deprivation: attachment mediates effects of caregiving on psychopathology. J Am Acad Child Adolesc Psychiatry.

[33] Atkinson L, Paglia A, Coolbear J, Niccols A, Parker KC, Guger S (2000). Attachment security: a meta-analysis of maternal mental health correlates. Clin Psychol Rev.

[34] Goodman SH, Gotlib IH (1999). Risk for psychopathology in the children of depressed mothers: a developmental model for understanding mechanisms of transmission. Psychol Rev.

[35] Gunnar MR, Quevedo KM (2007). Early care experiences and HPA axis regulation in children: a mechanism for later trauma vulnerability. Prog Brain Res.

[36] Rincón-Cortés M, Sullivan RM (2014). Early life trauma and attachment: immediate and enduring effects on neurobehavioral and stress axis development. Front Endocrinol 5.

[37] Shankaran S, Lester BM, Das A, Bauer CR, Bada HS, Lagasse L, Higgins R (2007). Impact of maternal substance use during pregnancy on childhood outcome. Semin Fetal Neonatal Med.

[38] Miller BA, Smyth NJ, Mudar PJ (1999). Mothers’ alcohol and other drug problems and their punitiveness toward their children. J Stud Alcohol.

[39] Walsh C, MacMillan HL, Jamieson E (2003). The relationship between parental substance abuse and child maltreatment: findings from the Ontario Health Supplement. Child Abuse Neglect.

[40] Nordsletten AE, Larsson H, Crowley JJ, Almqvist C, Lichtenstein P, Mataix-Cols D (2016). Patterns of nonrandom mating within and across 11 major psychiatric disorders. JAMA Psychiatry.

[41] Jokiranta-Olkoniemi E, Cheslack-Postava K, Sucksdorff D, Suominen A, Gyllenberg D, Chudal R, Leivonen S, Gissler M, Brown AS, Sourander A (2016). Risk of psychiatric and neurodevelopmental disorders among siblings of probands with autism spectrum disorders. JAMA Psychiatry.

[42] Magee SR, Bublitz MH, Orazine C, Brush B, Salisbury A, Niaura R, Stroud LR (2014). The relationship between maternal-fetal attachment and cigarette smoking over pregnancy. Matern Child Health J.

[43] Kalland M, Sinkkonen J, Gissler M, Meriläinen J, Siimes MA (2006). Maternal smoking behavior, background and neonatal health in Finnish children subsequently placed in foster care. Child Abuse Negl.

[44] Gold AB, Keller AB, Perry DC (2009). Prenatal exposure of rats to nicotine causes persistent alterations of nicotinic cholinergic receptors. Brain Res.

[45] Kane VB, Fu Y, Matta SG, Sharp BM (2004). Gestational nicotine exposure attenuates nicotine-stimulated dopamine release in the nucleus accumbens shell of adolescent Lewis rats. J Pharmacol Exp Ther.

[46] Müller KU, Mennigen E, Ripke S, Banaschewski T, Barker GJ, Büchel C, Conrod P, Fauth-Bühler M, Flor H, Garavan H, Heinz A, Lawrence C, Loth E, Mann K, Martinot J, Pausova Z, Rietschel M, Ströhle A, Struve M, Walaszek B, Schumann G, Paus T, Smolka MN (2013). Altered reward processing in adolescents with prenatal exposure to maternal cigarette smoking. JAMA Psychiatry.

[47] Takiguchi S, Fujisawa TX, Mizushima S, Saito DN, Okamoto Y, Shimada K, Koizumi M, Kumazaki H, Jung M, Kosaka H, Hiratani M, Ohshima Y, Teicher MH, Tomoda A (2015). Ventral striatum dysfunction in children and adolescents with reactive attachment disorder: functional MRI study. BJPsych Open.

[48] Rivkin MJ, Davis PE, Lemaster JL, Cabral HJ, Warfield SK, Mulkern RV, Robson CD, Rose-Jacobs R, Frank DA (2008). Volumetric MRI study of brain in children with intrauterine exposure to cocaine, alcohol, tobacco, and marijuana. Pediatrics.

[49] Shimada K, Takiguchi S, Mizushima S, Fujisawa TX, Saito DN, Kosaka H, Okazawa H, Tomoda A (2015). Reduced visual cortex grey matter volume in children and adolescents with reactive attachment disorder. NeuroImage: Clin.

[50] Crow JF (2000). The origins, patterns and implications of human spontaneous mutation. Nat Rev Genet.

[51] Kong A, Frigge ML, Masson G, Besenbacher S, Sulem P, Magnusson G, Gudjonsson SA, Sigurdsson A, Jonasdottir A, Jonasdottir A, Wong W, Sigurdsson G, Walters GB, Steinberg S, Helgason H, Thorleifsson G, Gudbjartsson DF, Helgason A, Magnusson OT, Thorsteinsdottir U, Stefansson K (2012). Rate of de novo mutations, father’s age, and disease risk. Nature.

[52] Perrin MC, Brown AS, Malaspina D (2007). Aberrant epigenetic regulation could explain the relationship of paternal age to schizophrenia. Schizophr Bull.

[53] Hare EH, Moran PA (1979). Raised parental age in psychiatric patients: evidence for the constitutional hypothesis. Br J Psychiatry.

[54] Hinkka-Yli-Salomäki S, Banerjee PN, Gissler M, Lampi KM, Vanhala R, Brown AS, Sourander A (2014). The incidence of diagnosed autism spectrum disorders in Finland. Nord J Psychiatry.

[55] Susser E, Schwartz S, Morabia A, Bromet EJ (2006). Psychiatric epidemiology.

[56] Sarkola T, Kahila H, Gissler M, Halmesmäki E (2007). Risk factors for out-of-home custody child care among families with alcohol and substance abuse problems. Acta Paediatr.

[57] Jokiranta E, Brown AS, Heinimaa M, Cheslack-Postava K, Suominen A, Sourander A (2013). Parental psychiatric disorders and autism spectrum disorders. Psychiatry Res.

